# Intersecting Sexual and Reproductive Health and Disability in Humanitarian Settings: Risks, Needs, and Capacities of Refugees with Disabilities in Kenya, Nepal, and Uganda

**DOI:** 10.1007/s11195-015-9419-3

**Published:** 2015-10-23

**Authors:** Mihoko Tanabe, Yusrah Nagujjah, Nirmal Rimal, Florah Bukania, Sandra Krause

**Affiliations:** Women’s Refugee Commission, 122 East 42 Street, New York, NY 10168-1289 USA; Refugee Law Project, Plot 7 and 9 Perryman Gardens, Old Kampala, P.O. Box 33902, Kampala, Uganda; Association of Medical Doctors of Asia, Shital Marg, Maharajgung, Kathmandu, Nepal; International Rescue Committee, Kakuma Refugee Camp, Kakuma, Kenya

**Keywords:** Sexual and reproductive health, Disability, Refugees, Humanitarian settings, Kenya, Nepal, Uganda

## Abstract

The current literature recognizes the fact that persons with disabilities have historically been deprived of their sexual and reproductive health (SRH) rights. Little is known, however, about the situation for women, men, and adolescents with disabilities in humanitarian settings. The Women’s Refugee Commission led a participatory research project with partners to explore the risks, needs, and barriers for refugees with disabilities to access SRH services, and the practical ways in which these challenges could be addressed. The study gathered information from refugee women, men, and adolescents aged 15–19 with physical, intellectual, sensory, and mental impairments in refugee settings in Kenya, Nepal, and Uganda. Findings showed that refugees with disabilities demonstrated varying degrees of awareness around SRH, especially regarding the reproductive anatomy, family planning, and sexually transmitted infections. Among barriers to accessing services, lack of respect by providers was reported as the most hurtful. Pregnant women with disabilities were often discriminated against by providers and scolded by caregivers for becoming pregnant and bearing children; marital status was a large factor that determined if a pregnancy was accepted. Risks of sexual violence prevailed across sites, especially for persons with intellectual impairments. The ability of women with disabilities to exercise their SRH rights was mixed. Refugees with disabilities showed a mixed understanding of their own rights in relationships and in the pursuit of opportunities. Findings speak to the need to realize the SRH rights of refugees with disabilities and build their longer-term SRH capacities.

## Introduction

Article 25 (a) of the 2006 Convention on the Rights of Persons with Disabilities (CRPD) articulates that persons with disabilities should have the same range, quality, and standard of free or affordable health care and programs as provided to other persons. This includes sexual and reproductive health (SRH) services [1].

The current literature recognizes that persons with disabilities have historically been denied their SRH rights [2]. They may have less access to SRH information, which can lead to low levels of knowledge about HIV/AIDS and sexually transmitted infections (STIs), and high-risk behaviors [3]. They may also access SRH services less frequently, despite their being as sexually active as their non-disabled peers [4, 5]. Further, persons with disabilities may be subjected to forced sterilization, abortion, and marriage due to long-standing stigmatization [6]. Mistaken beliefs that persons with disabilities are asexual or hypersexual also increase their exposure to abuse and subsequent health consequences [7].

In humanitarian settings, the published and gray literature show that the marginalization and exclusion of persons with disabilities put them at increased risk of sexual violence, rape, domestic abuse, and physical assault [8–11]. Reported restrictions that limit their access to health services include non-accessible physical infrastructure, lack of suitable and affordable transportation, lack of assistive devices, and long wait times [12–14].

Efforts to reach persons with disabilities with SRH education and services primarily originate from non-humanitarian contexts [15]. Guidelines and tools have been developed around disability accessible health care, including disability-inclusive SRH programs [6, 16–18]. Nonetheless, few programs actively address the SRH needs of persons with disabilities in humanitarian settings. The needs of persons with disabilities are also notably absent from the standard guidance for SRH in emergencies, which does not address equitable SRH access for women, girls, boys, and men with disabilities [19].

## Background

To address this information gap, the Women’s Refugee Commission (WRC) led a participatory research project to examine the intersections between SRH and disability in the humanitarian contexts of Kenya, Nepal, and Uganda. The three sites were selected based on their varied displacement contexts, geographic diversity, and availability of SRH services. The study explored the specific risks, needs, and barriers for persons with disabilities to access SRH services, and the capacities and practical ways through which the challenges could be addressed.

As per the CRPD, “persons with disabilities” were defined as those who have “long-term physical, mental, intellectual, or sensory impairments which, in interaction with various barriers, may hinder their full and effective participation in society on an equal basis with others” [1]. “Barriers” explored were environmental, attitudinal, and structural. “Sexual and reproductive health,” as defined by the International Conference on Population and Development (ICPD), includes maternal and newborn health, family planning, STIs, including HIV, and gender-based violence [20].

The study in Kenya was conducted in November–December 2013 in partnership with the International Rescue Committee (IRC), in Kakuma Refugee Camp, which is home to refugees from 13 countries. At the time of the study, 2084 of the 128,560 refugees in the camp were registered as having disabilities, representing 1.6 % of the total population. This number is far below the 15 % of the global population that the World Health Organization (WHO) estimates are living with disabilities; this is due to limited means of identifying persons with disabilities in humanitarian settings [21].

In Nepal, the study was undertaken in August 2014 among Bhutanese refugees in Beldangi Refugee Camp (I, II, and Extension). The study was hosted by the Association of Medical Doctors of Asia-Nepal (AMDA), in coordination with the United Nations High Commissioner for Refugees (UNHCR), and in partnership with the Nepal Disabled Women Association, the National Federation of the Disabled Nepal, and the Damak Disability Helping Committee. As of August 2014, 25,433 refugees were residing in the camps; in March 2014, 854 were registered to have disabilities, representing approximately 3.4 % of the camp population.

The study in Uganda was undertaken with the Refugee Law Project (RLP) in Kampala in December 2013–January 2014. The urban capital is host to more than 46,000 refugees from neighboring countries. According to UNHCR, as of June 2013, 452 refugees with disabilities were registered in Kampala.

## Methodology

### Study Participants

The target populations selected for this study were refugee women of reproductive age (20–49 years), men (20–59 years), and adolescent girls and boys (15–19 years) with physical, intellectual, sensory, and mental impairments. In addition, caregivers and family members were consulted; priority was given to those who were caring for adolescents or adults with disabilities.

### Activities

The study used qualitative, participatory methods in group and individual formats. Based on a literature review and consultative processes with the study’s local and global advisory groups, questions explored knowledge of the reproductive system and fertility; barriers and challenges to accessing SRH services; perceptions of services; impact of stigma and caregiver/provider attitudes; protective strategies; and capacities and resources to meet SRH needs and protect from SRH risks. To assess these domains, the selected participatory activities for groups included body mapping, timelines, and sorting [22]. In keeping with existing guidelines and recommendations on disability inclusion [23], activities were adapted with visual aids, simple language, and other modifications.

Family members/caregivers were consulted through focus group discussions and interviews if they were unable to leave their homes due to caregiving responsibilities.

### Sampling and Segmentation

The overall study design employed a maximum variation approach to include different groups of refugees with disabilities. Persons with disabilities were divided by sex and age into four categories of refugees with:

1. Physical, vision, and mild mental impairments
2. Hearing impairments
3. Mild intellectual impairments
4. Other needs and impairments (those unable to leave their homes, those with multiple impairments, etc.)

Participants were divided by their ability to functionally communicate with other participants and the facilitator. No official assessment was undertaken to verify or “diagnose” an impairment, and participants were invited to self-identify their disability. Sign language was provided where participants had hearing impairments, while activities for participants with mild intellectual impairments were simplified as necessary. Individual interactions were used for persons with multiple disabilities, new mothers, and other persons for whom in-depth activities at a person’s home were more appropriate.

Different study instruments were used for group and individual activities; these were field-tested in each setting. The individual interview guide did not directly touch upon sensitive SRH issues in consideration of the presence of caregivers who often supported communications. Instead, interviews provided an opportunity to explore broader health topics and concerns.

For family members/caregivers, standard approaches to qualitative research for focus group size (6–12) and number were applied where feasible [24]. The same focus group discussion guide was used as the interview guide for caregivers who were unable to leave their homes.

In total, 287 refugees with disabilities participated in the study, of whom 185 were women and girls and 102 were men and boys. Sixty-five caregivers and family members were consulted. Tables 1 and 2 show the numbers of participants across sites.

**Table 1:** Number of participants across sites, by sex and age

	Women of reproductive age (20–49 years)	Men (20–59 years)	Adolescent girls (15–19 years)	Adolescent boys (15–19 years)	Caregivers/family members	Total
Kenya	41	23	20	11	17	112
Nepal	40	29	10	10	15	104
Uganda	50	17	24	12	33	136
Total	131	69	54	33	65	352

**Table 2:** Number of participants across sites, by impairment group

	1. Refugees with physical, vision and mild mental impairments	2. Refugees with hearing impairments	3. Refugees with mild intellectual impairments	4. Other refugees (home-based, new mothers, etc.)
Kenya	60	15	11	9
Nepal	30	38	16	5
Uganda	70	3	24	6
Total	160	56	51	20

Given challenges in discerning between impairment types and the focus on functional ability to communicate, some groups were mixed and/or included persons with multiple impairments (such as physical and hearing impairments)

### Participant Recruitment

Participants were recruited via existing lists, and through agency and community social connections. In Kenya, IRC community health mobilizers recruited refugees with disabilities from their respective blocks in the camp, who then provided additional contacts. In Nepal, UNHCR and Caritas Nepal recruited refugees with disabilities from existing program lists (the WRC conducted stratified sampling) and their networks, through home visits or cell phone calls. In Uganda, data collectors recruited participants via phone calls and home visits from contact lists managed by RLP, the Association for Refugees with Disabilities in Uganda, and the Somali community leader, as well as via snowball sampling. In all sites, staff or data collectors explained the broad purpose of the activities and expectations. An informational flier noting objectives, expectations, and use of findings was made available to potential participants in all languages employed in the study (see Table 3), including in Braille in Nepal.

**Table 3:** Refugee origins and languages employed in study, by site

Country	Refugee origins	Study languages
Kenya	Somalia, South Sudan, Ethiopia, Democratic Republic of Congo (DRC), Sudan, and Burundi	Somali, Kiswahili, Arabic, English, and Somali sign
Nepal	Bhutan	Nepali and Nepali sign
Uganda	Rwanda, Burundi, DRC, Sudan, Somalia, Ethiopia, and Eritrea	Swahili, Somali, Kinyarwanda, and Luganda sign

### Study Team Composition and Training

The WRC and co-investigators recruited 12 data collectors in each site. In Kenya, data collectors were refugee youth; in Nepal, persons with disabilities from collaborating organizations of persons with disabilities and their assistants; and in Uganda, refugee youth and adults, some of whom had physical disabilities. The data collectors participated in a 3.5–4-day training on human subjects research; SRH topics; communications skills; facilitation and recording; consent/assent processes; ethical data handling; and referral pathways. The trained data collectors piloted the study instruments and tools, and received frequent support and review of skills throughout data collection, particularly during daily debriefing sessions. Team members ultimately comprised facilitators, notetakers, and sign interpreters, as well as participant mobilizers and supporters in Uganda and Nepal, respectively.

### Informed Consent

Informed consent was sought from all refugees with disabilities in their local language and tailored to accommodate different impairments. Verbal consent was sought in Nepal and Uganda, while written consent was sought in Kenya, the latter per local institutional review board instructions. Languages for consent included all languages in which the study was conducted, including the respective sign language (see Table 3). The consent process included information on how participants were selected, the nature of the study, and the types of questions they would be asked if they consented. Participants were assured that individual names would not be collected or used in any study findings. Only those participants who consented were permitted to participate.

Potential participants who did not have capacity to provide full informed consent due to age or barriers in communication were asked to provide verbal agreement, and the caregiver was asked to provide permission. Per national laws, minors (15–17 years) were asked to assent, and a parent/guardian was asked to provide permission. Pregnant girls, those who had children, or those who were married or living on their own provided their own consent.

For persons with perceived intellectual impairments in particular, the consent/assent process was interactive to facilitate more effective communication and establish understanding of their involvement in the activities. As applied in other SRH-related studies [25], once objectives and the process had been explained, potential participants were asked four questions. If they answered two incorrectly, but still expressed interest in participating, caregiver/family member permission was sought. The same threshold applied to persons with other types of impairments.

During the time of the actual activity, onsite consent was obtained. The consent process was similar to the advance consent process, although specific ground rules, such as confidentiality and how to uphold it, were discussed. For activities among persons with intellectual impairments, the facilitator asked six interactive questions to capture understanding, and potential participants were required to answer three of the questions correctly. A “yes” to participate needed to be obtained from every person.

Caregivers/family members who participated in activities were asked only to provide verbal consent. The studies were approved for implementation in Kakuma by the Kenya Medical Research Institute, in Damak by the Nepal Health Research Council, and in Kampala by the Uganda National Council for Science and Technology.

### Other Ethical Considerations

Individuals were informed of existing health or psychosocial services if they revealed recent experiences of violence or requested additional information and services. Participants (and any accompanying caregivers) were reimbursed for transportation costs where applicable.

Personal identifiers were collected only for recruitment purposes. During data collection, no personal identifiers were recorded or retained. Mappings and timelines were later photographed for data analysis. Partner organizations collected the data collectors’ notes at the end of data collection activities. Typed transcripts were made available only to the WRC and co-investigator staff involved in the data analysis.

### Data Analysis

Daily debriefings were held with the study team. Team members reviewed responses to each question and directly translated their notes for the WRC and co-investigators to type notes in English. In all three sites, the WRC or the co-investigators facilitated an end-of-activity discussion with the data collectors to provide a forum to gather their perspectives.

The WRC analyzed the comprehensive notes with NVivo 10 and Excel. Findings were analyzed within and among activities, and comparisons made across site, sex, age, language, and impairment group, where feasible.

## Limitations

Not all impairments and ages were adequately represented, especially adolescents and persons with mental and intellectual impairments. In Nepal, resettlement had impacted participant availability, and in Uganda, signing participants were underrepresented due to the lack of a common sign language. The identification of persons with intellectual and mental disabilities was also challenging, as a strict screening process was not employed, and they were sometimes hidden from public view. Additionally, given that groups were segmented based on participants’ functional ability to communicate, some groups had spill-overs from other categories, especially if they had more than one impairment. Analysis thus focused on general and common findings rather than attempting to solicit saturation by impairment group or spoken language.

Social desirability bias may have been present in Kenya and Uganda, the former where IRC staff were involved in data collection, and the latter where some participants were RLP’s direct beneficiaries. The teams were trained to maintain a neutral and encouraging environment to minimize possible effects.

The use of various forms of sign language contributed to challenges across sites. In Nepal, some SRH terms—such as menstruation—did not exist in Nepali sign. Only information that could be assured some level of certainty was reported across sites.

Facilitated translation techniques were used in all sites where notes were typed immediately after the activity with the data collection team [26]. This minimized recall bias; however, translation errors and minor omissions due to data collector language capacity may still be present.

## Findings

### Awareness of SRH Concepts

In all three settings, refugees with disabilities demonstrated varying degrees of awareness around SRH, especially regarding the reproductive anatomy, family planning, and STIs. HIV and condom use for HIV prevention was most widely known across countries, age, sex, language, and impairment group. Overall, adolescent girls and boys tended to know less than adults. However, in Kenya, adolescents with access to schooling opportunities—including signing participants—generally had better knowledge of reproductive organs and their functions. Lack of awareness about SRH was most apparent among refugees with limited access to information, especially those with intellectual impairments in all three settings, as well as those isolated to their homes in Uganda. Among home-based participants in Uganda, one participant was not familiar with the notion of sexual intercourse, and only one participant was aware of the concept of family planning through her knowledge that pills existed to prevent pregnancy.

Family planning methods most frequently cited across countries were short-term methods (condoms, pills, and injectables). In Kenya, among refugees who were unable to leave their homes, those who were familiar with HIV and STIs had heard of at least one contraceptive method. No participant mentioned male and female sterilization. In Nepal, women who were using family planning methods were generally more familiar with options to space births, although participants across age, sex, and impairment group were much less aware about the intrauterine device and the female condom. No participant had heard of emergency contraception despite its availability in the camp. In Uganda, while between one and several participants in all groups could name at least one contraceptive method, mistrust and misconceptions were noticeable around family planning options. Many feared that condoms could get stuck inside a woman’s body, cause disease, or make a woman lose her fertility.

In terms of STIs, boys and men in Kenya appeared to know less than girls and women, who could cite one or two symptoms. This trend was also observed among groups that used sign language. In Nepal, despite one or two adults in each group listing names of STIs—primarily syphilis and gonorrhea—participants had little awareness about actual signs and symptoms. In Uganda, awareness appeared comparatively better, with most group participants able to list some symptoms, including painful urination, vaginal discharge, abdominal pain, and genital itching.

Familiarity with post-rape care varied across sites. While some group participants in Kenya—including adolescents—and a handful in Uganda were aware of the benefits of seeking health care after experiencing sexual violence, participants in Nepal were unaware of this care.

Despite awareness gaps, persons with disabilities across age, sex, language, and impairment group showed much interest in learning more about SRH. This was common across the three countries.

### Experiences Around Accessing or Using Health and SRH Services

In terms of perceptions around existing health/SRH services, positive feedback was overwhelmingly received in Nepal. The majority of refugees with disabilities in Nepal reported receiving good quality services at the AMDA camp clinic. Many said that staff attitudes had improved tremendously over the past one and a half years due to active changes made by AMDA. A sizeable number of refugees with disabilities and caregivers in Kenya also reported that they were satisfied with existing health services in the camp. A Swahili-speaking caregiver, for example, mentioned that most of the time they “receive good care and are treated equally like any other person.”

Despite positive feedback, a greater number of refugees with disabilities and their caregivers in Kenya and, especially, Uganda complained about challenges to accessing health services. Negative and disrespectful provider attitudes were reported as the most influential barrier that deterred refugees with disabilities from accessing services. In Kenya, one Somali caregiver, explained, “In hospitals, we face a lot of pressure. They [health providers] belittle us because we have disabled patients.” In Uganda, negative provider attitudes were a critical problem at health centers and the national referral hospital. An adult male participant in a Swahili-speaking physical, vision, and mental impairment group shared, “Health workers think persons with disabilities do not have a right to sex, yet they are also normal like other people.” Refugees with intellectual impairments also stated: “[Persons with disabilities] are overlooked and neglected by doctors and nurses,” and “[Health providers] don’t consider them like normal human beings.”

Other reported barriers included long wait times (Kenya and Uganda), costs to seeking care (Uganda), refugee status (Uganda), communication with providers (all three sites), caregiver and community attitudes (Uganda), lack of transportation (Kenya and Uganda), and limited accessibility (all three sites). In Uganda, where all aforementioned concerns were raised, refugees with disabilities and caregivers listed as barriers to accessing care: the lack of translation, for both spoken and sign language; lack of transportation to health facilities; limited wheelchair availability at the referral hospital; stock-outs of medicines; and lack of money to pay health providers. Many agreed that if they did not have money, due to their refugee status and the added disadvantage linked to disability, they would be largely ignored by health providers. Some mentioned that they would wait all day to receive services: “Sometimes, we go at 07:00, and we come home at 18:00. That is tiresome when there is nothing done for us.” Most groups mentioned that “if you are disabled, you wait, wait, wait.” Related comments around language barriers—especially the lack of sign language interpreters—were similarly noticeable in Kenya and Nepal.

### Experiences Around Pregnancy for Women and Girls with Disabilities

Participants generally agreed that if a girl or woman with disabilities became pregnant, her marital status would determine how she would be treated. If she were married, the pregnancy would be welcome. This attitude was widespread across sites, sex, age, language, and disability category.

On the other hand, if the girl or woman with disabilities was not married, participants across sites and segmented categories agreed that she would experience serious discrimination. Refugees with disabilities felt that the family and neighbors would say she is “a prostitute,” that she had “misbehaved,” or that she “was raped.” In Uganda, where criticism was harshest, Somali-speaking participants felt pregnancy out of wedlock would be problematic due to their culture, although Swahili-speaking male groups with intellectual impairments also attested to possible beatings if the girl or woman was not married. In Nepal, both marital status and existence of a disability appeared to impact the way pregnant women and girls with disabilities were treated, although, similar to the other two countries, marital status seemed the greater factor.

Indeed, many group participants in Nepal—especially women—often associated pregnancy out of wedlock as a result of sexual violence, rather than of romantic relationships: “If a girl with a disability is not married but is pregnant, people will think she was raped.” Women with hearing impairments were identified as being particularly at risk of sexual violence. In all three sites, risks of sexual violence were raised for persons with intellectual impairments, especially for adolescent girls. Caregivers in Uganda shared: “When the girl has an intellectual problem, when she goes out, other men can take advantage of her, like raping her.”

Despite pregnancy in marriage appearing to be more welcome in all three settings, pregnant women and girls with disabilities were still subject to remarks from caregivers. Caregivers in Uganda were reportedly concerned about the additional responsibilities they would assume when their family member bore children. For example, when people around a Swahili-speaking woman with a physical disability discovered that she was pregnant, the mother-to-be noted: “Others were happy and others were not happy since I am disabled and yet I am pregnant. How will I care for my baby?…They couldn’t believe I could become pregnant.”

The incredulity was reflected in provider attitudes in Uganda, especially during the birthing process. Many participants felt that pregnant women and girls with disabilities would not be treated nicely and with respect by health providers. They cited receiving remarks such as: “How can you as a refugee and disabled person be pregnant?” and said: “Discriminated by the midwives, the nurses would mock her because she is a problem and she is giving birth to another problem.” On the other hand, most participants in Kenya and Nepal agreed that the woman who was giving birth would be treated nicely and with respect by health providers, reflecting different experiences across sites.

### Experiences Around Romantic Relationships and Unions

While participants in Kenya and Uganda treated adolescents with disabilities having romantic relationships as natural in a person’s life course, a high level of mistrust around relationships was observed among adolescent girls in Nepal who noted that they could be “cheated” due to their disability. Indeed, several adult women with disabilities in Nepal explained, “In the case of disabled, there are lots of cases where the husband will leave, divorce, or hate.” In Nepal, disability was mentioned as a source of violence risk in marital relationships. Moreover, in Nepal and Uganda, several women with disabilities were observed to have less stable relationships and were subsequently caring for children without a partner, raising protection concerns.

### Ability of Refugees with Disabilities to Exercise SRH Rights

The ability/autonomy of women with disabilities to exercise their SRH rights was mixed across and within groups in the three sites. For unmarried women and girls who find themselves pregnant, participants in the three sites offered a range of possibilities, including: hide the pregnancy; run away from home; commit suicide (Nepal); keep the pregnancy; abort the pregnancy; be forced to abort the pregnancy; be forced to marry; or choose to marry the baby’s father and raise the child together. The possibility of forced abortion was most strongly observed among the Somalis in Uganda. Somali caregivers cited reasons such as: “While abortion is not allowed in our religion, we would go ahead and do that to save ourselves from blame,” as well as, “It is our reputation that will be tarnished, so we will get rid of the baby.” In these instances, however, the decision to terminate a pregnancy was largely based on marital status and less on disability.

To prevent future unplanned pregnancies, participants also cited a spectrum of possibilities, ranging from full autonomy to none. In Kenya, Somali adolescents felt: “The woman can decide for herself. It is a partner decision—the man and the woman together.” Despite some suggestive liberal thinking, participants across language, age, and sex also voiced more restrictive consequences. For example, Arabic-speaking female participants suggested, “The parents will take her to hospital and tell the clinicians to give her any family planning method to avoid her becoming pregnant again, with or without her consent.” Somali adolescent boys with mild intellectual impairments additionally suggested, ‘“She will be locked in the house,” and “She might be circumcised again.” Such feedback suggests limited autonomy in SRH decision-making, especially for unmarried women and adolescent girls with disabilities.

Comparable ranges in possibilities were shared by participants in Uganda: Swahili speakers tended to note that “they decide for themselves because the parents do not advise their girls on the methods to use.” The Somalis more often mentioned that families, especially the mothers would be involved: “To protect her, we would do this [give her pills, injections, or an intrauterine device], with or without her consent.” Only one group—Swahili-speaking women with mild intellectual impairments—defended refugees with disabilities and their ability to have subsequent pregnancies, claiming: “No one can stop because it is her right. Although she is a person with a disability, she has a right to produce.”

In Nepal, to prevent unplanned pregnancies, most participants felt the decision was up to the woman, man, or the couple. Contrary to the other two sites, all groups demonstrated openness regarding contraceptive choice, irrespective of marital status.

### Understanding of Rights by Refugees with Disabilities Themselves

To gauge the level of understanding that refugees with disabilities possessed around their own rights, 25–28 cards were developed with pictorial scenarios and accompanying text for participants to sort into categories of “acceptable,” “unacceptable,” or possibly “both” (see Table 4). All participants agreed that violence against persons with disabilities—especially sexual violence—was unacceptable. Among the three sites, refugees with disabilities in Kenya were most aware of their rights. Much more variation was observed in Nepal and Uganda, where “forcing a person with a disability to be sterilized” and “controlling money” received the most mixed responses.

**Table 4:** Categorizations of acceptable and unacceptable scenarios by site, sex, and age

	Overall	Kenya	Nepal	Uganda	Female	Male	Women	Girls	Men	Boys
*Sexual violence*										
Rape of an adult	2	2	2	2	2	2	2	2	2	2
Rape of a child	2	2	2	2	2	2	2	2	2	2
Sexual harassment	2	2	99	99	2	2	2	2	2	2
Sexual exploitation and abuse	3	2	2	3	3	2	3	2	2	2
Forced prostitution	2	2	2	2	2	2	2	2	2	2
Molestation	2	99	2	2	2	2	2	2	2	2
*Physical violence*										
Beating of an adult with a disability by a family member	2	2	2	2	2	2	2	2	2	2
Beating of a child with a disability	3	2	2	3	3	2	3	2	2	2
Neglect	2	2	2	2	2	2	2	2	2	2
Forcing a PWD to be sterilized	3	2	3	3	3	3	3	3	3	2
Denying access to services	3	2	2	3	3	2	3	2	2	2
Child labor	3	2	3	3	3	2	3	3	2	2
*Emotional violence*										
Violence with words	3	2	3	2	3	2	3	2	2	2
Making a PWD see traumatic acts	2	2	2	2	2	2	2	2	2	2
Rejecting or abandoning the PWD	2	99	99	2	2	3	2	2	3	2
*Economic violence*										
Controlling money	3	2	3	3	3	3	3	3	3	3
Not allowing opportunity	2	2	2	2	2	2	2	2	2	2
Human trafficking	2	99	2	2	2	2	2	2	2	2
Low or no payment for work	2	2	2	2	2	2	2	2	2	2
*Harmful traditional practices*										
Early marriage	3	3	2	3	3	3	3	2	2	3
Female genital cutting	3	2	99	3	3	2	2	3	2	2
Child sacrifice	3	99	99	3	3	2	3	2	2	2
Promoting traditional or cultural myths about PWDs	3	2	2	3	3	2	3	3	2	2
*Non-violence*										
Non-violent, happy family where PWDs are included	1	1	1	1	1	1	1	1	1	1
PWDs and non-PWD adolescents are friends	3	1	3	1	1	3	1	1	3	1
PWDs in safe, happy romantic relationships	3	1	3	3	3	3	3	3	3	1
Someone offering help to a PWD	1	1	1	1	1	1	1	1	1	1
A PWD child attending mainstream school	3	1	3	3	3	3	1	3	3	1
A PWD as a leader of a community	3	1	3	3	3	3	3	3	3	3

*1* Acceptable, *2* unacceptable, *3* both, *99* excluded from site

Regarding the former, when examining by sex and age, all groups but adolescent boys felt there were possible justifications around sterilizing a person with a disability. In Uganda, 25 % of groups noted that forced sterilization could be acceptable, with comments including: “When a person has a [intellectual] problem, the caretaker is the one to decide. If the person has a physical impairment, the person could produce. If the person has a heavy disability and is disturbing the family, they may have the person sterilized.” In Uganda, all adolescent groups categorized forced sterilization as unacceptable; counter responses came only from adults. Across the three sites, adolescent boys unanimously agreed that forced sterilization was unacceptable.

“Controlling money” was the most contested of all scenarios, and this was seen across sex and age groups in Nepal and Uganda. In Uganda, participants often stated that it was acceptable to control the money of persons with intellectual or visual impairments, although such claims were not echoed by persons with the said impairments. For example, adult Somali women with mild intellectual impairments in Uganda said, “Controlling money is unacceptable because persons with disabilities have a right to have their money to give to whom they want.”

Six seemingly positive scenarios were also provided to participants. Results from the three countries showed that only two of the six scenarios received unanimous votes as “acceptable” (see Table 4). When compared across sites, participants in Kenya agreed that all six scenarios were acceptable; when compared across sex and age, adolescent boys most agreed to the acceptability of the six scenarios.

Among those who felt “Persons with disabilities in safe, happy, romantic relationships” to be “unacceptable” or “both” in Nepal, participants again referred to the possibility that they could be cheated. In Uganda, aside from one group of Swahili-speaking women with mental impairments, participants agreed that persons with disabilities have, “a right to love.”

In terms of the scenario “Persons with disabilities and persons without disabilities are friends,” an adult male signing participant in Nepal felt, “Deaf persons will always be discriminated, and thus not accepted.” Even the scenario “A child with disabilities attending mainstream school” was regarded negatively by signing men, who agreed that they do “not accept, because the children with hearing impairments can’t learn with the other students without sign language.”

The scenario “A person with a disability as a leader of a community” received mixed responses in Nepal and Uganda, with comments reflecting attitudes of refugees with disabilities around their own capacities. In Nepal, men and adolescent boys with hearing impairments felt, “There is no good communication, then the leader will be cheated, so this is not acceptable.” In Uganda, negative feedback included, “She can’t be a leader because she is disabled and no one will respect her.”

In group activities, the degree of acceptable touching was probed to some extent to examine awareness around safety risks. In Kenya, one 16-year-old Somali adolescent girl with physical impairments shared, “It is play if a person can touch you anywhere.” When probed, it appeared the girl had been raised to believe this. In Nepal, adolescent girls with intellectual impairments were not always aware of the difference between appropriate and inappropriate touching.

### Protective Resources and Facilitating Factors

While physical and sexual risk factors prevail for refugees with disabilities, participants across sites mentioned protective resources that offered emotional and mental respite. In Kenya, an English-speaking adult woman with multiple impairments said that she is a choir member and is happy when she hears other people sing during church service. Others mentioned having “peace of mind” when community members, religious persons, or friends visit and pray for them. In Uganda, persons with mental disabilities reported RLP’s counselors as safe and reassuring. Several caregivers felt schools—when safe—were a protective space for their children, as interactions with other children and the acquisition of communication skills improved their home situation. In both Uganda and Nepal, home-based refugees reported family members, especially mothers, as safe resources. While self-help activities were on the whole limited, participants across sites noted their ability and willingness to share information with each other and with other refugees and leaders in the community.

### Recommendations from Refugees with Disabilities and Caregivers

Recommendations offered by refugees with disabilities to improve their SRH experience often reflected ways to improve their care experience, as well as activities to empower themselves. Suggestions included training providers on respectful communication skills; employing sign language and other interpreters in health facilities; expanding SRH awareness activities; reducing wait times for services; and receiving peer learning, leadership, skills building, and income-generation opportunities. In Uganda, participants also requested resettlement options.

## Discussion

It is well documented that the SRH rights of persons with disabilities are frequently overlooked and often violated [6]. The CRPD marks a significant shift in recognizing the rights of persons with disabilities to make their own informed decisions about all issues that affect them, including their sexuality and reproduction, and to live free from violence, discrimination, and coercion [27]. This study demonstrates, however, that refugees with disabilities do not have the same access to SRH information and services as others in their communities.

Common findings show that awareness is influenced by level of access to external sources of information. In Kenya, school-going adolescents and signing participants were familiar with SRH topics. Likewise in Nepal, where AMDA had provided SRH messaging, awareness across sex, age, and impairment group was apparent. However, findings reveal an overall gap in SRH awareness, pointing to the need to provide SRH information to adolescents and parents with disabilities. Including persons with disabilities in SRH activities is expected to decrease the awareness gap between refugees with different types of impairments and increase opportunities for home-based refugees to receive information from external sources. These efforts will help refugees with disabilities realize their right to a healthy sexuality [28].

In terms of barriers to accessing health and SRH services, attitudes—especially those of the provider—appeared to be the predominant and most hurtful. The mentioned barriers speak to the need to implement provider training on communicating with refugees with disabilities in a respectful manner, and understanding and appreciating the SRH rights of persons with disabilities. Other barriers, such as the lack of sign interpreters in health facilities, lack of transportation, and long wait times have been documented by other studies; operationalizing the CRPD calls for making reasonable accommodations [1, 13].

The study found that in all three sites, marital status was a greater determinant than disability in how pregnant women and girls with disabilities would be treated. A pregnancy was welcome in the context of marriage, while extra-marital pregnancies were heavily disapproved of. Pregnant women with disabilities in Uganda were also reported to experience discrimination in the birthing process. The incredulity around the possibility that women with disabilities can become pregnant mirrors prevailing notions that mistakenly assume persons with disabilities to be asexual or without equal rights to bear children as able-bodied persons [7].

Findings further show the varying degrees of autonomy that refugees with disabilities have in exercising their SRH rights, as well as the mixed understanding of their own rights as persons with disabilities. Possibilities of forced abortion, forced use of family planning, or forced sterilization violate the fundamental human rights of persons with disabilities, which are further protected in the CRPD. SRH and disability rights education for refugees with disabilities, their families, service providers, and the community at large is crucial to upholding the human rights of all.

Mixed attitudes among some groups of persons with disabilities and caregivers toward other persons with disabilities reflect social prejudices. This was most prominent in Uganda, where persons with physical impairments were observed to show unequal attitudes towards those with intellectual impairments, especially around forced sterilization. Such thoughts reflect wider societal attitudes toward persons with intellectual impairments with respect to their autonomy over exercising SRH rights, including the choice to use or not to use contraception or terminate a pregnancy.

The ability of refugees with disabilities to exercise their rights was in some instances curtailed by low self-esteem, especially in Nepal and Uganda. Such situations can benefit from increasing access to leadership skills, disability rights knowledge, sexuality education, peer interaction, vocational training, and income-generation opportunities, to foster empowerment and longer-term SRH capacities.

Protection concerns, including sexual violence, were apparent for adolescent girls with intellectual impairments, in particular. This group could be reached through targeted awareness-raising that focuses on acceptable touching, contraceptive choice, protection from violence, and information on when to seek help or services. At the same time, the availability of emergency contraception and post-exposure prophylaxis can be introduced to all refugees with disabilities as part of the conversation around post-rape care and the importance of seeking timely medical care after sexual assault.

Other noticeable protection issues include single mothers with disabilities who lack support in parenting, especially in Nepal and Uganda. Their circumstances raise concerns about abuse and exploitation in and outside of the family. Women with disabilities may benefit from additional support in raising children, as well as community-based protection mechanisms to foster and monitor their inclusion.

Despite prevailing risks, participants mentioned protective resources, especially persons and activities that offer emotional and mental respite. Increasing the engagement of refugees with disabilities in social functions, as well as their contact with safe social networks, is expected to enhance their protection and increase their outlets for information sharing and learning [8]. Both Nepal and Uganda have well appreciated organizations of refugees with disabilities—such fora can be further supported to ensure that refugees with disabilities have a voice in community decision making, as well as social support to reduce risks and enhance their empowerment and long-term well-being.

## Conclusion

Findings from the study speak to the need to protect and realize the SRH rights of refugees with disabilities. A practical first step can be to foster disability inclusion in SRH services through training staff; budgeting for disability inclusion; and enhancing outreach, protection, and engagement. Programs can further offer opportunities to refugees with disabilities around leadership skills, disability rights knowledge, sexuality education, peer interaction, vocational training, and income generation to build longer-term SRH capacities and address their overall empowerment. Targeted outreach and emphasis to meet the SRH needs of refugees with disabilities can further the rights of this resilient group.
